# Surgical Technique for Complementing Robotic-Assisted Total Knee Arthroplasty in Middle-Aged Patients with Rigid Knee Bones

**DOI:** 10.3390/jpm14090945

**Published:** 2024-09-06

**Authors:** Ji-Hoon Baek, Su Chan Lee, Suengryol Ryu, Dong Nyoung Lee, Taehyeon Kim, Hye Sun Ahn, Chang Hyun Nam

**Affiliations:** Joint & Arthritis Research, Department of Orthopaedic Surgery, Himchan Hospital, Seoul 07999, Republic of Korea; jihoon011@naver.com (J.-H.B.); himchanhospital@naver.com (S.C.L.); oszzang102@naver.com (S.R.); fretless@hanmail.net (D.N.L.); ysunispica@naver.com (T.K.); ahs0614@naver.com (H.S.A.)

**Keywords:** robotic-assisted, total knee arthroplasty, complementary technique

## Abstract

This study reports 12 cases of inaccurate bone cutting from a single-surgeon series of 509 consecutively performed robotic-assisted total knee arthroplasty (TKA) for 1 year. In addition, a complementary technique with the combined benefits of robotic-assisted and manual techniques that address this issue is described. From June 2020 to May 2021, a consecutive series of 509 TKAs was performed on 338 patients using a posterior-stabilized total knee prosthesis with a robotic-assisted system at our hospital. The surgical records were reviewed to identify the causative bone locations and bone re-cutting events correlated with improper trial instrument positioning. The re-cutting rate was 2.4% (12/509). All re-cutting attempts occurred because of improper cutting of the femoral posterior chamfer. Re-cutting was attempted mostly on middle-aged male patients. This complementary technique can facilitate manual bone cutting while retaining the advantages of robotic surgery during robotic-assisted TKA. Additionally, the combined technique of manual bone cutting and robotic-assisted surgery can be a useful alternative for middle-aged male patients with rigid knee bones.

## 1. Introduction

The use of robotic-assisted techniques for primary total knee arthroplasty (TKA) provides the benefits of accurate bone resection, appropriate component positioning, restoration of mechanical alignment, and early improvements in short-term clinical outcomes compared with conventional techniques [1,2,3,4,5]. However, robotic systems also have disadvantages that are not found in conventional TKA. Notably, these disadvantages are associated with pin tracker- and longer surgical duration-related complications [6,7]. These disadvantages are attributed to the additional steps performed by the robotic system, which include the insertion and removal of pin trackers in the femur and tibia, registration of the knee joint with the robotic system, and intraoperative planning [6]. Several studies have attempted to overcome these limitations and have achieved good results [8,9,10].

A robotic system (MAKO) was introduced at our hospital in June 2020. MAKO (Stryker, Kalamazoo, MI, USA) is the most representative semi-active robotic system [11]. It can determine the appropriate implant size and location based on preoperative computed tomography (CT) images of the extremities. The method employs additional mapping using a handheld probe during the operation to construct a surface map of the knee and match it with the preoperative CT data. The MAKO robotic arm with haptic feedback demonstrates a feature in which sawing is discontinued when the pre-set parameter exceeds a value during bone cutting [12]. Cutting the femur and tibia via robotic-assisted TKA is performed using a saw blade controlled by the robotic system equipped with the robotic arm. A total of six parts are cut using the robotic arm saw: distal femur, anterior femur, anterior chamfer, posterior chamfer, posterior condyle (in the femur), and proximal tibial (in the tibia). After a year of robotic surgical experience, a new disadvantage that had not been reported in the literature was identified. In some patients, certain parts of the femur were inaccurately cut using the robotic arm saw. In other words, the posterior chamfer was cut inaccurately during the femoral bone cutting process in some patients with rigid knee bones. Consequently, because of imprecise cutting, there was imprecise fitting between the bone and the trial instrument when inserting the trial femoral instrument, resulting in multiple re-cutting attempts. Thus, some cases require modifications depending on the experience of the surgeon performing the surgery in addition to the surgical techniques offered by the robotic system. Subsequently, we devised an instrument and technique that enabled the easy manual cutting of hard bone parts. Herein, we report 12 cases of inaccurate bone cutting from a single-surgeon series of 509 consecutive robotic-assisted TKA procedures performed over a period of 1 year. In addition, a complementary technique with the combined benefits of robotic-assisted and manual techniques that addresses this issue is described.

## 2. Materials and Methods

The Institutional Review Board of our institution approved the design and protocol of this retrospective study. The need for informed consent was waived (IRB number: 116655-01-202202-01). From June 2020 to May 2021, a consecutive series of 509 TKAs was performed on 338 patients using a triathlon posterior-stabilized total knee prosthesis with a robotic-assisted system (MAKO) at our hospital. Patients with preoperative Kellgren–Lawrence grade IV osteoarthritis, rheumatoid arthritis, or osteonecrosis were included. Demographic data, including age, sex, body mass index, initial diagnosis, operative time, and total blood loss, were obtained by reviewing medical records. The operative time ranged from the initial skin incision to the final wound closure. Total blood loss was calculated using the Gross equation [13].

All primary robotic-assisted TKAs conducted during the study period were included in the analysis. All surgeries were performed by a single high-volume knee surgeon. The preoperative goals included achieving neutral alignment and a polyethylene liner thickness of 9 mm. TKAs were performed using the standard medial parapatellar approach. The surgical records of the patients were reviewed to identify the causative bone locations and bone re-cutting events correlated with improper trial instrument positioning. The robotic-assisted surgical technique, design of the new chamfer cutting guide, and complementary surgical technique are described below.

Student’s *t*-test was used to analyze age, body mass index, operative time, and total blood loss. Fisher’s exact test was used to examine sex, and the chi-square test was used to examine diagnosis. The analyses were conducted using the Statistical Package for the Social Sciences software version 18.0 (IBM Inc., Armonk, NY, USA). All reported *p*-values were two-sided, and a *p*-value < 0.05 was considered statistically significant.

### 2.1. Robotic-Assisted Surgical Technique

**Figure 1:**
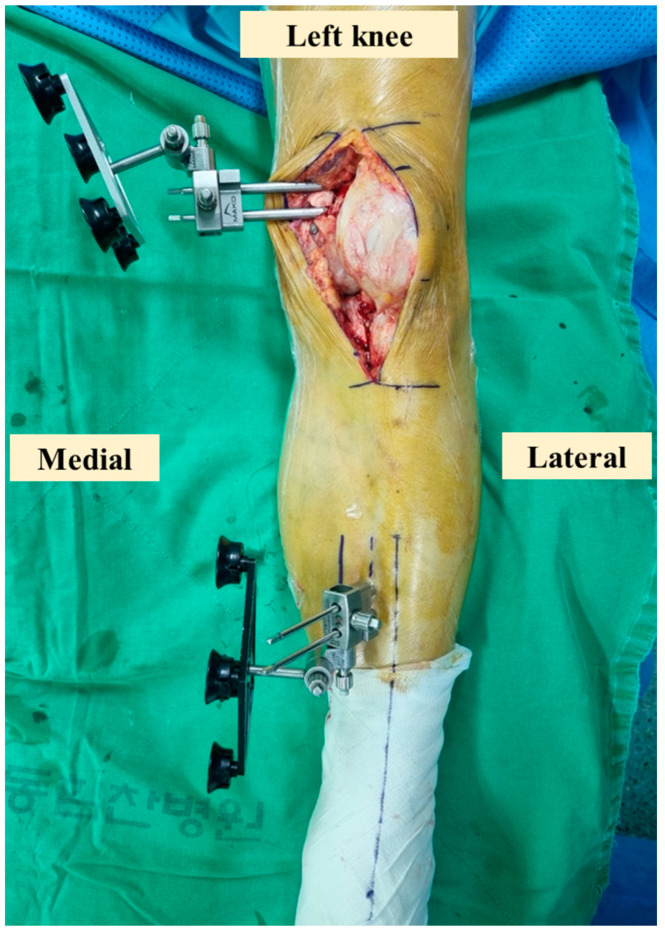
Anterior view of the femoral and tibial pin trackers. The femoral pin tracker was placed on the medial side of the distal femur, and the tibial pin tracker was placed approximately 20 cm inferior to the knee joint line in the diaphysis.

Before the robotic-assisted TKA procedure, a preoperative CT scan of the lower limbs was performed. The results were incorporated into the robotic system’s software to identify the optimal implant size, alignment, and positioning for the patient’s knee. In the operating room, all knees were exposed using the standard medial parapatellar approach while sacrificing the anterior and posterior cruciate ligaments. None of the patients’ patellae were replaced, and only osteophytes were excised. Pin trackers [8] and checkpoints were positioned on the tibia and femur (Figure 1). Landmark calibration and bone registration were performed using a probe to identify the real bone position and intraoperative coronal alignment. Intraoperative ligament and gap balancing were performed manually with spoons or a lamina spreader via knee mechanics, including knee flexion, varus/valgus, and rotation. Appropriate adjustments were made to the robotic software to balance and gap symmetrically. Appropriate implant positions and orientations were defined and saved in the robotic system after the surgeon’s approval.

Resection of the distal femur and posterior chamfer was performed using a robotic arm angled saw. Resection of the anterior femur, anterior chamfer, posterior condyle, and proximal tibia was performed using a straight saw within the virtual boundaries defined by the robot to protect the soft tissues. After femoral box cutting, the femoral and tibial trial implants and the liner thickness were assessed. If the medial and lateral portions of the trial implants had poor bone fitting, or if the trial implants were in a flexion or extension position, an inadequate bone cut was considered, and re-cutting was attempted using a robotic arm saw. When the cutting was considered appropriate after multiple re-cutting attempts, the femoral and tibial implants were placed using bone cement, and a polyethylene liner was inserted. All patients received a posterior-stabilized triathlon total knee prosthesis (Stryker Orthopedics), and all implants were inserted using bone cement.

### 2.2. New Chamfer Cutting Guide Design

**Figure 2:**
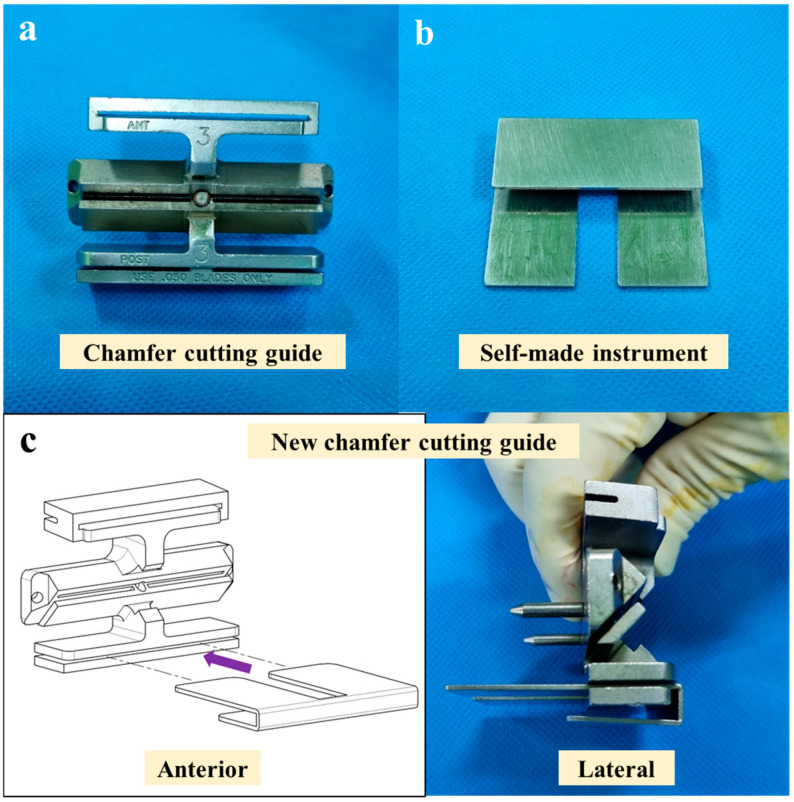
(**a**) Chamfer cutting guide for conventional Stryker triathlon total knee arthroplasty. (**b**) A self-made instrument that can contact the resected posterior condyles of the femur. (**c**) A new chamfer cutting guide comprising the existing chamfer cutting guide (**a**) and the self-made instrument (**b**).

The new chamfer cutting guide (Figure 2) was devised to manually cut the anterior femoral bone and the anterior and posterior chamfers of the femoral bone when using the complementary technique. The new chamfer cutting guide comprises a combination of the chamfer cutting guide (Figure 2a) and a self-made instrument (Figure 2b). The chamfer cutting guide is an instrument used to cut the femoral bone in conventional Stryker triathlon TKA, and the self-made instrument was custom-made. When the self-made instrument plays the role of a support fixture by fitting into the resected posterior condyles of the femur, the chamfer cutting guide can be fixed on the resected surface of the distal femur. Subsequently, a manual saw can be used for femoral bone cutting.

### 2.3. Complementary Surgical Technique

The complementary robotic-assisted TKA technique has been used in our hospital for middle-aged male patients with rigid knee bone, according to the surgeon’s preference, since June 2021. The process from preoperative CT scan, preoperative planning, and joint exposure to intraoperative planning was the same as the surgical process described above.

**Figure 3:**
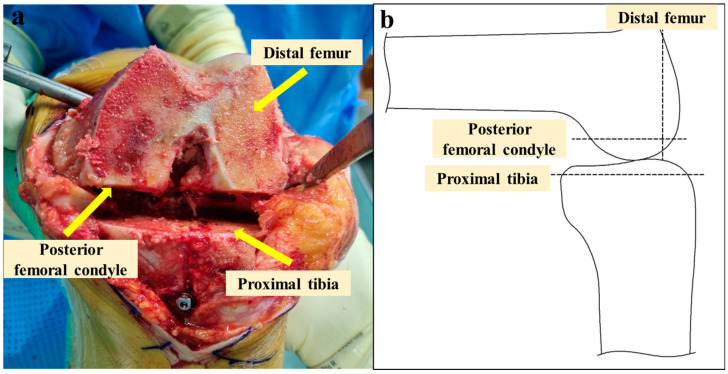
(**a**) Resected bone appearance of the distal femur, posterior femoral condyle, and proximal tibia. (**b**) Simple lateral illustration of (**a**).

**Figure 4:**
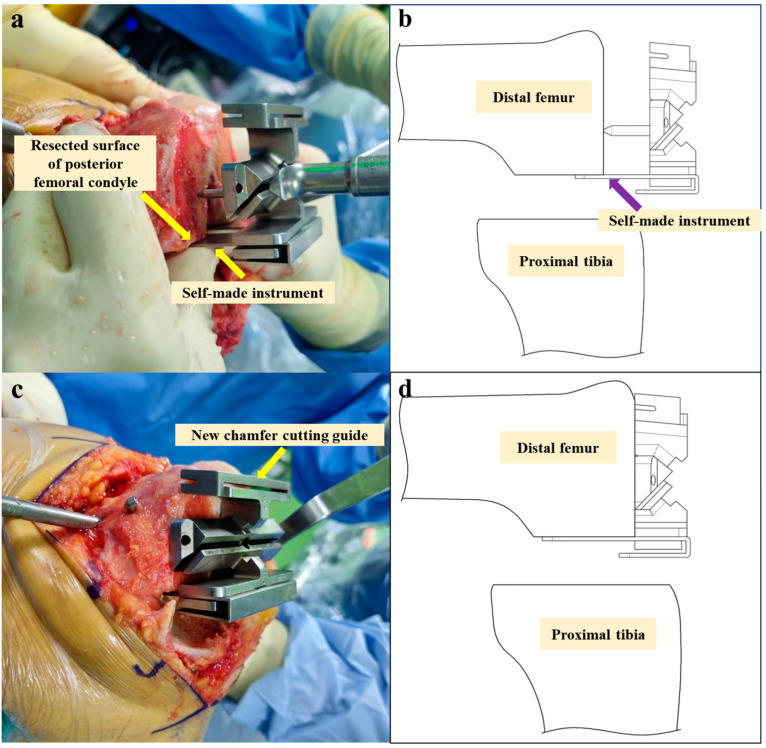
(**a**) The self-made instrument was placed against the resected posterior condyles of the femur. (**b**) Simple lateral illustration of (**a**). (**c**) New chamfer cutting guide attached to the resected distal femur surface with the feet in contact with the resected posterior condyles of the femur. (**d**) Simple lateral illustration of (**c**).

**Figure 5:**
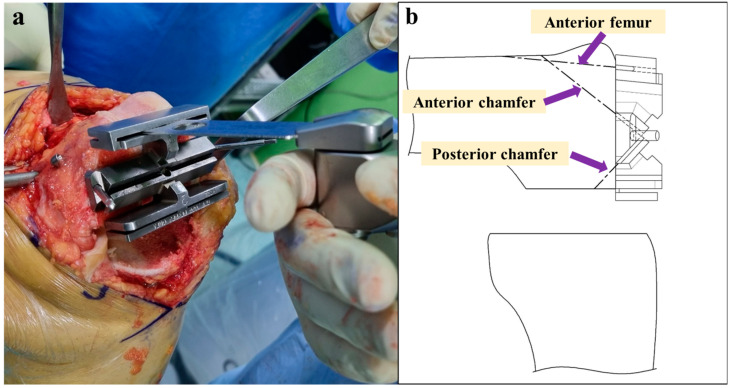
(**a**) The remaining three bone cuts, including the anterior femur, anterior chamfer, and posterior chamfer, were manually resected using an oscillating saw blade. (**b**) Simple lateral illustration of (**a**).

Three bone cuts—distal femur, posterior femoral condyle, and proximal tibia—were performed within the virtual boundaries set by the robotic system to protect the soft tissues (Figure 3). The new chamfer cutting guide (Figure 2) was placed flat against the resected surface of the distal femur, with the feet in contact with the resected posterior condyles of the femur (Figure 4). After fixing the chamfer cutting guide and removing the self-made instrument, the remaining three bone cuts—anterior femur, anterior chamfer, and posterior chamfer—were manually performed using an oscillating saw blade (Figure 5). After femoral box cutting, the femoral and tibial trial implants, as well as the thickness of the liner, were assessed. Knee alignment, ligament balance, and knee kinematics were assessed. The femoral and tibial implants were placed using bone cement, and a polyethylene liner was inserted.

## 3. Results

All robotic-assisted primary TKAs performed during the 1-year study period were analyzed. The mean patient age at the time of TKA was 70.9 (range: 53–87) years, and the study population included 48 men (65 knees) and 290 women (444 knees). Initial diagnoses included primary osteoarthritis in 498 knees (n = 327), rheumatoid arthritis in 10 (n = 10), and osteonecrosis in 1 (n = 1).

Of the 509 TKA procedures, 12 (n = 11) involved re-cutting. All re-cutting attempts occurred because of improper cutting of the femoral posterior chamfer. The re-cutting rate was 2.4% (12/509).

The re-cutting group included 10 male patients (11 cases) and 1 female patient (1 case), and their average age at the time of primary TKA was 62.2 (range: 54–67) years. Non-re-cutting group included 38 men (54 cases) and 327 women (443 cases), and their average age at the time of primary TKA was 71.2 (range: 53–87) years. The mean operative time was longer in the re-cutting group than in the non-re-cutting group (60.8 ± 12.0 min vs. 53.5 ± 10.4 min, *p* < 0.05). There was no significant difference in total blood loss between the re-cutting and non-re-cutting groups (586.7 ± 134.1 mL vs. 577.9 ± 131.4 mL, *p* = 0.819). The clinical information of the re-cutting group was compared with that of the non-re-cutting group (Table 1). The two groups differed in terms of sex, age, and operative time.

## 4. Discussion

During femoral bone cutting using a robotic arm saw, inappropriate cutting may occur in the posterior chamfer in middle-aged male patients with hard knee bones. This may cause improper femoral trial implant fitting, leading to multiple re-cutting attempts. This study demonstrated that this complementary technique can be used to manually cut hard bones with ease while maintaining the advantages of robotic surgery.

Robotic-assisted TKA techniques are novel alternatives to conventional TKA [14,15]. This technology can lead to more accurate component positioning and limb alignment and has several distinct advantages over conventional TKA [1,2,3,16]. However, robotic-assisted techniques have some disadvantages, and efforts have been exerted to overcome them recently. An example of such efforts concerns complications associated with pins. In particular, pin tract-induced periprosthetic fracture, which does not occur in conventional procedures, can be encountered after robotic-assisted procedures. Therefore, the pin position was changed to prevent periprosthetic fractures related to the pin site position [8]. In this study, inadequate cutting typically occurred in middle-aged male patients with a rigid bone during femoral posterior chamfer cutting using a robotic arm saw, resulting in multiple re-cutting attempts. The re-cutting rate was 2.4%, which is not a small percentage considering the increasing use of robotic surgery [17,18]. In addition, the operative time was longer in the re-cutting group than in the non-re-cutting group because the former had a higher proportion of male participants, had younger participants, and had undergone multiple cutting attempts because of a hard bone. Therefore, a surgical instrument that can accurately cut rigid bones, such as the posterior chamfer of the femur, while maintaining the advantages of robotic-assisted TKA was developed. This complementary technique has been used in our hospital for middle-aged male patients with rigid knee bones, according to the surgeon’s preference, since June 2021. Intraoperatively, a rigid bone may be decided, especially in middle-aged male patients, when the cartilage of the medial femoral bone has worn away so much that the bone is exposed and the surface has hardened due to years of friction with the tibial bone. In this case, when cutting the bone with an angled saw, the posterior chamfer bone may be improperly cut; therefore, a complementary technique is used.

**Figure 6:**
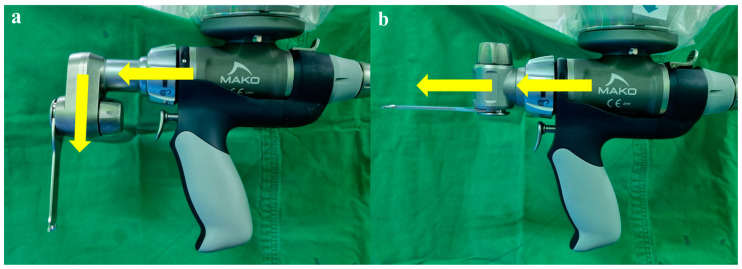
(**a**) Angled saw: electric power was transmitted by bending 90° into the angled saw. (**b**) Straight saw: electric power was directly transmitted to the straight saw.

When cutting the bone using the MAKO system, a total of six parts were cut using the robotic arm saw. Two cuts were made to the distal femur and posterior chamfer using an angled saw (Figure 6a). Furthermore, four cuts were made to the anterior femur, anterior chamfer, posterior femoral condyle, and proximal tibia using a straight saw (Figure 6b). Improper bone cutting may occur in all six bone parts and can result in an improper fit between the bone and the trial implant. In our study, performing these four cuts with a straight saw was possible, even if the bones were hard. However, in a few middle-aged male patients with hard bones, the angled saw failed to cut the bones accurately. In particular, improper bone cutting occurred in the posterior chamfer bone rather than the distal femur. This can be assumed in two cases: First, the cutting force of an angled saw may be weaker than that of a straight saw. This is because the electric power was transmitted by bending 90° into the angled saw. Second, there is less surface area for the angled saw to rub against the bone in the posterior chamfer bone than in the distal femur, so it can be assumed that the saw is less likely to encounter cancellous bone in hard bone, resulting in a poor cut.

In robotic surgery, the femoral and tibial implant positions were set in advance before bone cutting. After cutting the distal bone, femoral posterior condyle, and proximal tibial bone using the robot system, cutting the remaining bones manually did not affect the surgical plan of the robotic-assisted surgery. Using the complementary technique, the resection of the hard posterior chamfer bone was performed without any issues when manual bone cutting was performed using an oscillating saw blade. A self-made instrument was made to use this complementary technique and used in addition to the chamfer cutting guide used in the conventional technique to cut the remaining bone (Figure 2). This combination technique can be used to facilitate hard bone resection while maintaining the advantages of the robotic-assisted technique.

In summary, this complementary technique is applicable in two cases. The first is if the surgeon believes that the knee bone is likely to be rigid. First, the robotic arm saw was used to cut the distal femur, posterior condyle, and proximal tibial bones, and the remaining bone cuts were manually cut using the new chamfer cutting guide. The second condition was inadequate trial femoral implant fitting after all six bone cuts were performed using a robotic arm saw. First, re-cutting with the robotic arm saw is attempted, and then the implant trial is attempted again. If an inadequate trial femoral implant fitting still occurs, manual re-cutting should be attempted using the new chamfer cutting guide. After four years of experience since the introduction of the robot in our hospital, the author has also found the second method useful during robotic surgery.

This study has several limitations. First, the procedure was conducted at a single center, and all procedures were performed by a high-volume robotic knee surgeon. Therefore, the generalizability of the results may be limited. Second, the proposed technique is applicable to specific robotic-assisted systems. However, the MAKO system is currently the most commonly used intelligent surgical robot for joint surgery worldwide [19]. Third, the number of patients requiring this technique (n = 12) was low, which might have limited the justification for using this method. Similar to assisted navigation and customized cutting guides, surgeons should occasionally perform some cuts manually. Hence, this technique is also useful for complementing bone cutting during robotic-assisted TKA.

## 5. Conclusions

A total of six parts were cut during TKA using the MAKO robot: the distal femur, anterior femur, anterior chamfer, posterior chamfer, posterior condyle bone in the femur, and proximal tibia bone in the tibia. In particular, when cutting the posterior chamfer bone using the robotic arm angled saw, several excision attempts may be required due to improper bone cutting in some middle-aged male patients with hard knee bones. However, this complementary technique can facilitate manual bone cutting while retaining the advantages of robotic surgery during robotic-assisted TKA. The combined technique of manual bone cutting and robotic-assisted surgery can be a useful alternative for middle-aged male patients with rigid knee bones.

## Figures and Tables

**Table 1:** Demographic characteristics of the patients.

	Re-Cutting Group	Non-Re-Cutting Group	*p*-Value
Number of patients	11	327	
Number of cases	12	497	
Sex (male)	10/11 (91.0%)	38/327 (11.6%)	<0.05
Age (years) (mean ± SD)	62.2 ± 4.2 (54–67)	71.2 ± 6.2 (53–87)	<0.05
Body mass index	25.8 ± 3.0 (23.0–31.6)	26.2 ± 3.4 (18.7–37.3)	0.757
Diagnosis			1.000
Osteoarthritis	12 (100%)	486 (97.8%)	
Rheumatoid arthritis	0	10	
Osteonecrosis	0	1	
Operative time (min) (mean ± SD)	60.8 ± 12.0 (40–80)	53.5 ± 10.4 (30–85)	<0.05
Total blood loss (mL) (mean ± SD)	586.7 ± 134.1 (410–850)	577.9 ± 131.4 (300–850)	0.819

SD—standard deviation.

## Data Availability

The data presented in this study are available from the corresponding author upon request. The data are not publicly available due to privacy reasons.
